# A Case Report of Sporadic Adult Neuronal Intranuclear Inclusion Disease (NIID) With Stroke-Like Onset

**DOI:** 10.3389/fneur.2020.00530

**Published:** 2020-06-10

**Authors:** Pan Lin, Hang Jin, Kun-Chang Yi, Xiang-Sheng He, Shi-Fang Lin, Gang Wu, Zai-Qiang Zhang

**Affiliations:** ^1^Department of Neurology, The Second Hospital of Longyan City, Longyan, China; ^2^Neuroscience Centre, Department of Neurology, The First Hospital of Jilin University, Changchun, China; ^3^Department of Neurology, First Affliated Hospital of Fujian Medical University, Fuzhou, China; ^4^Department of Neurology, Fujian Sanbo Funeng Brain Hospital, Fuzhou, China; ^5^Department of Neurology, Beijing Tiantan Hospital, China National Clinical Research Center for Neurological Diseases, Capital Medical University, Beijing, China

**Keywords:** neuronal intranuclear inclusion disease, stroke, neurodegenerative disease, skin biopsy, FXTAS

## Abstract

**Background:** Neuronal intranuclear inclusion disease (NIID) is a rare neurodegenerative disease. The clinical manifestations of NIID are complex and easily misdiagnosed. Based on the current knowledge of this disease, it is usually chronic, with almost no acute cases. Stroke-like disease is an extremely rare type of NIID.

**Case Presentation:** A 61-year-old woman was admitted to our hospital with sudden left limb weakness. Diffusion magnetic resonance imaging (MRI) demonstrated high signal intensity in the skin-medullary junction area. Tissue pathology showed eosinophilic inclusions in the nuclei of the sweat gland cells and fat cells of the skin. Subsequent genetic analysis of the fragile X chromosome mental retardation gene 1 (*FMR1*) gene showed that the CGG repeat number was in the normal range, excluding fragile X-related tremor/ataxia syndrome (FXTAS). After 3 weeks of hospitalization, the patient's condition improved, and the left limb muscle strength recovered. Her symptoms were almost completely diminished after 3 months.

**Conclusion:** This case demonstrates the strong clinical heterogeneity of NIID. NIID can manifest as acute hemiplegia and a stroke-like attack. This case study provides new information for the diagnosis of NIID and the classification of the clinical characteristics.

## Introduction

NIID is a rare, slowly progressive neurodegenerative disease characterized by the presence of eosinophilic hyaluronan inclusions in the central and peripheral nervous system and internal organs. It is difficult to diagnose NIID only through clinical symptoms and signs, and as a result, this condition is often misdiagnosed. In the last century, NIID could only be diagnosed through invasive methods, including a nerve biopsy and necropsy. Since Sone et al. (1) found in 2011 that a skin biopsy could be used to diagnose NIID, the number of confirmed cases of NIID has increased.

The clinical manifestations of NIID are very complex, involving the central nervous system and peripheral nervous system, as well as non-nervous tissue, such as the kidney (2). NIID can commonly present as a chronic neurodegenerative disease but occasionally presents acutely. According to published case reports, acute NIIDs consist mainly of paroxysmal encephalopathy. Most patients present with sudden headache and fever as the first symptom (3), accompanied by a consciousness disorder or epilepsy (4). The above symptoms can also be recurrent (5, 6). However, we could not locate any reports of a stroke-like onset of hemiplegia in NIID. Recently, we encountered a case of a typical sporadic adult NIID patient with stroke-like onset in routine clinical practice. The first symptom in our patient was acute left hemiplegia, and MR imaging showed diffusion-weighted imaging (DWI) high signal intensity in the cortex medullary junction area. Finally, the diagnosis was confirmed by pathological examination of the patient's skin biopsy and genetic analysis. This case report describes a rare clinical manifestation of NIID, which is helpful to update the understanding of the clinical characteristics of NIID and provide a reference for future research.

## Case Presentation

**Figure 1:**
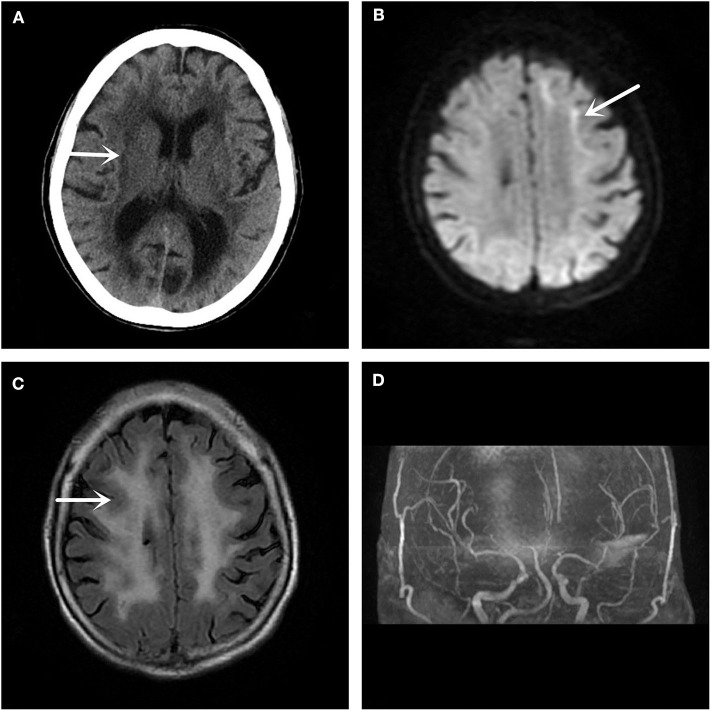
Imaging examination. **(A)** The head CT shows a low-density lesion in the white matter area. **(B)** The DWI sequence of the head MRI suggests a high signal shadow in the white matter and gray matter junction area, especially on the left side, which is characteristic of the inclusion disease in the nuclei of the neurons. **(C)** The T2 Flair sequence of the skull MRI also shows abnormal signals in the white matter region of the brain. **(D)** No significant intracranial aortic stenosis or occlusion changes are observed in the head MRA.

In December 2017, a 61-year-old woman was admitted to our hospital because she had been suffering from sudden left limb weakness for 3.5 h. She denied a history of smoking, high blood pressure, diabetes, and other cerebrovascular disease risk factors. She denied hereditary cerebrovascular history and other neurological genetic history. The clinical examination found that she had slurred speech, with a left limb strength of Grade 3, a positive left Babinski sign, and a National Institute of Health stroke scale (NIHSS) score of 6 points. An emergency computed tomography (CT) scan of her brain showed leukoaraiosis and senile brain changes (Figure 1A). An initial diagnosis of cerebral infarction was made, with indications for intravenous thrombolysis, and intravenous thrombolytic therapy was initiated with 40.5 mg rt-PA. Following thrombolysis, the patient's condition worsened the next day with new symptoms of lethargy, poor spirit, poor understanding, and the inability to move her left limbs. Additionally, there were new abnormal symptoms, including a bilateral positive Babinski sign accompanied by high fever, high body temperature between 38.5 and 39.3°C, but no headache, vomiting, limb convulsions, or incontinence. After active anti-thrombotic, anti-infective, anti-pyretic, and other treatments, the patient's condition stabilized. The MRI (Figures 1B,C) and magnetic resonance angiography (MRA) (Figure 1D) of the head showed improvement. The DWI (Figure 1B) was suspicious for “neuronal intranuclear inclusion disease,” and a skin biopsy of the right thigh was performed.

**Figure 2:**
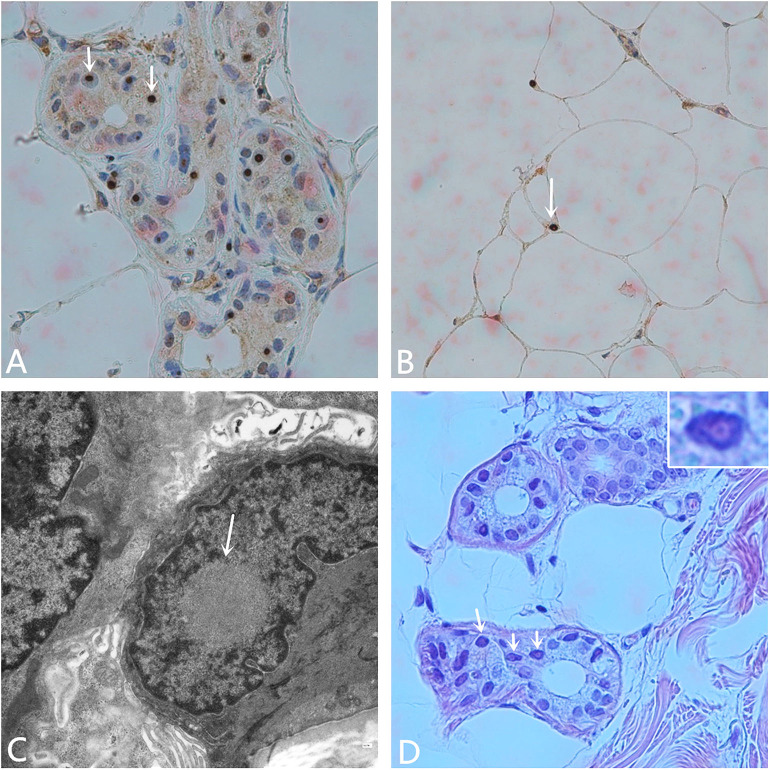
Pathological examination. **(A)** Anti-P62 immunohistochemical staining. Visible inclusion bodies in the nucleus of some skin sweat gland cells (× 400). **(B)** Anti-P62 immunohistochemical staining. Visible inclusion bodies in the nucleus of some fat cells (× 400). **(C)** Electron microscopy shows a circular inclusion body structure in the nucleus of a sweat gland (× 15,000). **(D)** Hematoxylin-eosin staining shows eosinophilic inclusion bodies in some sweat gland cells. The inset on the upper right shows amplification of one of the nuclei (× 400).

Other hospital-related examinations were carried out, including routine blood testing. The results showed a white blood cell count of 10.68 × 10^9^/L, a neutrophil ratio of 90.6%, C-reactive protein < 1.28 mg/L, procalcitonin 0.129 ng/ml, and an erythrocyte sedimentation rate (ESR) of 25 mm/h. Fasting blood glucose concentration 5.70 mmol/L, total cholesterol concentrations 4.66 mmol/L, low density lipoprotein concentration (LDL-C) 2.78 mmol/L, other biochemical indicators, routine urine and stool testing, glycosylated hemoglobin, tumor markers, thyroid function, five indicator test for Hepatitis B, human immunodeficiency virus antibody, the toluidine red non-heated serum test, and all other indicators were normal. Anti-nuclear, anti-double-stranded DNA, anti-SM, anti-SSB, anti-SSA, anti-JO-1, and all other autoantibody tests were negative. The Widal test and the Weil–Felix test were negative. A pharyngeal swab for bacterial and pathogen culture showed no abnormalities, and a lumbar puncture showed an intracranial pressure of 85 mmH_2_O. There were no abnormal findings in the cerebrospinal fluid cytological, biochemical, or bacteriological tests, or other indicators. No abnormalities were seen on vascular ultrasonography, echocardiography, or abdominal color Doppler ultrasound. Electromyography (EMG) showed that the bilateral ulnar nerve and common peroneal nerve conduction velocity were slightly slower than normal, and the shortest latency of the bilateral phrenic nerve F wave was prolonged. Subsequent analysis for the fragile X chromosome mental retardation gene 1 (*FMR1*) showed that the CGG repeat number was in the normal range, excluding fragile X-related tremor/ataxia syndrome (FXTAS). The pathology report of the skin biopsy (Figure 2) showed eosinophilic inclusions in the nuclei of the sweat gland cells and fat cells of the skin.

With characteristic image support, definitive pathological diagnosis, and optimal gene detection, the case was diagnosed as sporadic adult neuronal intranuclear inclusion disease. This patient experienced a stroke-like NIID, the first symptom of which was left limb weakness, followed by the gradual appearance of fever and a disturbance in consciousness. After 3 weeks of hospitalization, the patient regained consciousness, her fever subsided, and her left limb muscle strength recovered to Grade 4^+^. She could walk slowly. In March 2018, her symptoms were almost completely relieved. Her left limb muscle strength was close to normal, and there were no abnormal neuropsychiatric symptoms or signs.

## Discussion

Sone and colleagues summarized the clinical characteristics of 38 cases of sporadic NIID, most of which presented as the first and main clinical manifestations of dementia. Other clinical symptoms of sporadic NIID included consciousness disorders, limb weakness, sensory disorders, tremors, convulsions, behavioral abnormalities, and ataxia (7). In the aforementioned study, no cases of stroke-like acute onset were mentioned. Even if the first symptom of the patient is weakness of the limbs, it will progress slowly. Here, we reported a stroke-like case with hemiplegia of the left limb as the first symptom.

Because there was no specific diagnostic sign on emergency CT, this patient was considered to be a stroke victim at the ultra-early stage and received intravenous thrombolysis treatment. In this case, the patient's condition worsened after admission, and new symptoms, including a change in consciousness and fever, appeared. In addition, the positive Babinski signs on both feet indicated that the lesions might be located in the brainstem, so we considered whether this case was a post-circulation stroke. At this time, MRI is particularly important because it can confirm the location of the diagnosis. Unexpectedly, the MRI of the head showed that the focus was not in the posterior circulation, but in the anterior circulation of the corticomedullary junction area, with no macrovascular lesions on MRA. If it is not stroke, we suspected that it is an acute encephalitis, metabolic disease, or poisoning. With limited knowledge in neurodegenerative or NIID-related areas, we did not immediately recognize the characteristic imaging of NIID. This patient had a very small range of imaging lesions, and did not reconcile with the severity of her clinical symptoms. We consulted peers, searched the literature, and explained the case from the perspective of monism, with a likely diagnosis of NIID.

MRI showed DWI high signal intensity in the skin-medullary junction area, also known as the “cortical line sign,” a characteristic imaging manifestation of NIID (7, 8). Abe et al. (9) reported that over a 10-year period, imaging of a NIID patient showed that high signal intensity persisted in the DWI skin-to-medullary junction zone and progressed from the frontal lobe to the occipital lobe. This characteristic does not generally disappear throughout the course of the disease and can be an important marker for the diagnosis of NIID. Recently, some scholars found that the fragile X-related tremor/ataxia syndrome (FXTAS) also had similar features on imaging (10), and the clinical symptoms of the two are quite similar. Therefore, NIID should be distinguished from FXTAS by the number of CGG repeats in the *FMR1* gene (11). Along with the strong evidence from imaging and gene detection, we finally confirmed this case as NIID by pathological examination of the skin biopsy.

We observed that this patient with NIID developed a fever and a mild disturbance in consciousness after a stroke-like onset, which seems to be explained by the characteristics of paroxysmal encephalopathy. This is somewhat similar to a case reported by Tong (5), demonstrating that NIID may present differently in different stages. The EMG of this case also showed an abnormality of the peripheral nerve, implying that the central damage of the NIID patient was accompanied by hidden peripheral nerve damage. In summary, the clinical heterogeneity of NIID at different stages of the disease increased the difficulty and complexity of our diagnosis. This case provides an excellent example of the utility of diagnostic imaging in the diagnosis of rare diseases such as NIID. Only when we fully understand a disease are we able to diagnose it correctly.

Similar to other neurodegenerative diseases, NIID has no specific recommended treatment. At present, there are no appropriate methods to treat the pathophysiological mechanism of NIID, only supportive therapy and symptomatic treatment. In this case, NIID was treated with non-specific treatment, careful observation, and care. The symptoms of weakness, fever, and other symptoms gradually decreased after more than 3 months, and the patient's condition was temporarily improved. In the future, international studies with larger numbers of patients could elucidate the pathogenesis of NIID, enabling the development of more effective treatments for this condition.

## Data Availability Statement

The datasets analyzed in this article are not publicly available. Requests to access the datasets should be directed to 1192260154@qq.com.

## Ethics Statement

The patients/participants provided written informed consent for the publication of this case report.

## Author Contributions

PL and HJ wrote the report. K-CY and X-SH care for the patient. S-FL and GW performed the diagnostic testing. Z-QZ confirmed the pathological diagnosis. Written consent to publish was obtained from the patient.

## Conflict of Interest

The authors declare that the research was conducted in the absence of any commercial or financial relationships that could be construed as a potential conflict of interest.
